# Ten Simple Rules for Lifelong Learning, According to Hamming

**DOI:** 10.1371/journal.pcbi.1004020

**Published:** 2015-02-26

**Authors:** Thomas C. Erren, Tracy E. Slanger, J. Valérie Groß, Philip E. Bourne, Paul Cullen

**Affiliations:** 1 Institute and Policlinic for Occupational Medicine, Environmental Medicine and Prevention Research, University Hospital of Cologne, Cologne, Germany; 2 Office of the Director, The National Institutes of Health, Bethesda, Maryland, United States of America; 3 Medizinisches Labor Münster, Münster, Germany; Whitehead Institute, UNITED STATES

“A mathematician, like a painter or poet, is a maker of patterns. If his patterns are more permanent than theirs, it is because they are made with ideas.”—G. H. Hardy, “A Mathematician’s Apology” [1]

Learning is a lifelong imperative for any scientist, and Richard Hamming provided timeless advice on how to achieve this. In this sequel to our 2007 contribution to the Ten Simple Rules series [2], we attempt to distil the essence of what this mathematician and computer science and telecommunications pioneer addressed in one of his talks [3] and in his book *The Art of Doing Science and Engineering*: *Learning to Learn* [4]. Hamming developed both the talk and the book as a synthesis of his graduate course in engineering at the United States Naval Postgraduate School in Monterey, California. We have organized his authoritative advice into ten rules. We believe these will equip the reader to more confidently face the unremitting emergence of an exponentially increasing amount of new knowledge, coupled with the equally relentless obsolescence of established knowledge, in a world containing a greater number of scientists than ever before. Our rules promote a certain “style of thinking.” They also emphasize orientation towards the future and—we hope—will help the reader learn how to learn while motivating him or her to continue learning throughout life.

## Rule 1. Cultivate Lifelong Learning as a “Style of Thinking” That Concentrates on Fundamental Principles Rather Than on Facts

As Hamming indicates, learning to learn depends on a certain style of thinking [4]. An important distinction here is between education and training: education is learning what you should do and when and why to do it, whereas training is learning how to do it. Obviously, to succeed you need to be both educated and trained. But in this process, it is important not to be a slave to facts. In 1968, David Horrobin, who interacted with Karl Popper, Linus Pauling, and Sir John Eccles, wrote that “the so-called facts which the student learns are at least five years out of date when he meets them and after ten years of medical practice they will be almost dead” [5]. As John Hunter has said, “too many facts crowd the memory without advantage” [6]. Therefore, when faced with new knowledge, try to establish nodal points in the knowledge network; these will help you to reconstruct the essence of what you have learned even if you can’t remember the details. As Hamming said, “concentrate on fundamentals, at least what you think at the time are fundamentals, and develop the ability to learn new fields of knowledge when they arise so you will not be left behind” [4]. As you learn, watch out for “principles [6], “fundamentals” [4], and “patterns” (see the gem by Hardy [1] in the preface to this Editorial). These are likely to have much longer half-lives than the details involved. A further elaboration of this rule appears in [5]: “To paraphrase John Hunter, facts are important only in so far as they lead to principles: it is the principles which are vital and it is on these which you must concentrate. Fortunately, they are usually easier to remember than the facts themselves and also, once remembered, they allow the facts to be deduced from them”. In other words, it is important to understand what your knowledge network is made for rather than knowing what it is made of.

## Rule 2. Structure Your Learning to Ride the Information Tsunami Rather Than Drown in It

Exponential growth of the amount of knowledge is a central feature of the modern era. As Hamming points out, since the time of Isaac Newton (1642/3-1726/7), the total amount of knowledge (including but not limited to technical fields) has doubled about every 17 years. At the same time, the half-life of technical knowledge has been estimated to be about 15 years [4]. If the total amount of knowledge available today is x, then in 15 years the total amount of knowledge can be expected to be nearly 2x, while the amount of knowledge that has become obsolete will be about 0.5x. This means that the total amount of knowledge thought to be valid has increased from x to nearly 1.5x. Taken together, this means that if your daughter or son was born when you were 34 years old, the amount of knowledge she or he will be faced with on entering university at age 17 will be more than twice the amount you faced when you started college.

To put it differently, during an average working life, the amount of valid knowledge in any field can be expected to at least double, while more than three-quarters of what we think we know now will have become obsolete. This process may well continue indefinitely [4]. In view of the additional fact that the human brain can process data no faster than about 60 bits per second [7], it is clear that we need some kind of structure to our learning if we are to ride the information tsunami [8] rather than drown in it.

## Rule 3. Be Prepared to Compete and Interact with a Greater and More Rapidly Increasing Number of Scientists Than at Any Time in the Past

As Hamming points out, despite a levelling-off in some of the richer countries [9], the number of scientists worldwide continues to increase rapidly, and it has been estimated that about 90% of the scientists who ever lived are alive today. There is no reason to expect that this trend is about to change any time soon [10].

## Rule 4. Focus on the Future but Don’t Ignore the Past

As usual, Hamming put it trenchantly: “Teachers should prepare the student for the student’s future, not for the teacher’s past” [4]. Some teachers object to this, saying that no one can know the future. This is of course true but misses the point. We do not need to know what will happen—we just need to be ready when it does.

However, when riding the information tsunami into the future, try not to ignore the past entirely, as it may contain important information you may not be aware of. For example, an article in Nature under the headline “Where have I seen that before?” draws attention to the 103 years that elapsed between an experiment already done in 1908 and its accidental replication as the discovery of “new chemistry” in 206–2007 [11, 12].

## Rule 5. Look for the Personal Angle

Hamming uses anecdotes from his own life to highlight concepts about how to learn. Most of us do not move in exalted circles such as the research division of Bell Laboratories in Hamming’s time, but this need not prevent us from looking for the personal angle, private detail, or scientific feud that may help us to anchor the key concepts in our minds within a meaningful and significant context.

In that vein, we may infuse our learning with personal details, which some scientists publish. Vivid exchanges between Watson, Crick, and Chargaff during and after the double helix was discovered are an example of this. Watson documented that Chargaff was irritated when Crick found it hard to “remember the chemical differences among the four bases” [13] as a guide to which one-to-one ratios suggested that things were likely to go together. In 1976, Chargaff recalled meeting the Nobel laureates Watson and Crick in 1952:

…what I really was, when I first met the fervid pair in Cambridge, was baffled, for here were two people trying to fit the nucleotides into a helix and worrying about its pitch—it became a double helix, I believe, after I told them about our results—without bothering to look up the structures of the compounds they wanted to fit together [14].

Clearly, that Chargaff referred to his competitors inter alia as “two pitchmen in search of a helix” [14] (Pitchmen is an American term referring to hawkers such as those seen on TV shopping channels. These are always in search of some new “amazing discovery” to foist on a gullible public. Chargaff is also making a pun on the pitch of the double helix.) and as “scientific clowns” [13] provides a flavor of what can happen at the personal level in the “House of Science” [14]. Equally clearly, personal information can contribute to remembering details of the science involved.

## Rule 6. Learn from the Successes of Others

As Hamming says, because “there are so many ways of being wrong and so few of being right, studying successes is more efficient, and furthermore, when your turn comes you will know how to succeed rather than how to fail.” In addition, he notes that “vicarious learning from the experiences of others saves making errors yourself” [4].

To exemplify, there is much we can learn from the successes of Sir John Eccles. He was wrong in expecting that synaptic transmission was electrical rather than primarily chemical. However, Eccles was right when, inspired by Popper’s rigor to formulate testable hypotheses, he instigated experiments that ultimately contributed to falsifying the hypothesis of electrical synaptic transmission. Moreover, in 1963, Eccles was awarded a Nobel prize, together with Huxley and Hodgkin, for elucidating “the language of electrical nerve impulses and the responses of nerve cells engaged in replying to it at synapses” [15]. Taken together, Eccles could be viewed as an example that rigorous scientific reasoning and work qualify for the “few [ways] of being right” [4], irrespective of the hypothesis you investigate. Making this your work mode may yield the strongest recognitions for scientists, i.e., even the Nobel prize.

## Rule 7. Use Trial and Error to Find the Style of Learning That Suits You

Hamming says that learning how to learn is like learning how to paint. Both need advice but also individual trial and error:

How to be a great painter cannot be taught in words; one learns by trying many different approaches that seem to surround the subject. Art teachers usually let the advanced student paint, and then make suggestions on how they would have done it, or what might also be tried, more or less as the points arise in the student’s head—which is where the learning is supposed to occur! [4]

Your teachers are thus important, but do not be afraid to think for yourself. To cite Horrobin again, “perhaps the most important thing for a student to realize is that while his teachers may differ from him in experience, with rare exceptions they will not differ in intelligence. They are not individuals to fear, but individuals to question and challenge” [5].

## Rule 8. No Matter How Much Advice You Get and How Much Talent You Possess, It Is Still You Who Must Do the Learning and Put in the Time

To quote Hamming, “I am…only a coach. I cannot run the mile for you” [4]. You must test how Hamming’s advice in these ten rules works for you. You will only get out of these suggestions as much as you put in. Grapple with the issues involved and compare what is offered here with your own experience and that of others. Select and adapt those points that help you.

A prerequisite, of course, is native talent. But even for the talented, no amount of utilizing smart methods can substitute for sheer duration of effort. Gladwell has suggested that about 10,000 hours of application are needed to become a true expert in a particular field [16]. While some have quibbled with the universal validity of this suggestion, we think it is a fairly good estimate of what you need to put in. Also, these long hours need to be quality time without distractions. Easy to say, hard to do.

## Rule 9. Have a Vision to Give You a General Direction

A key to learning how to learn is to be economical and to structure your efforts according to the general direction in which you want to move. Hamming writes: “It is well known the drunken sailor who staggers to the left or right with *n* independent random steps will, on the average, end up about *√n* steps from the origin. But if there is a pretty girl in one direction, then his steps will tend to go in that direction and he will go a distance proportional to *n*.” [4]

You too need an attractive vision of where you want to go. As Hamming points out, “having a vision is what tends to separate the leaders from the followers.”

## Rule 10. Make Your Life Count: Struggle for Excellence

Overall, Hamming is “preaching the message that, with apparently only one life to live on this earth, you ought to try to make significant contributions to humanity rather than just get along through life comfortably—that the life of trying to achieve excellence in some area is in itself a worthy goal for your life.…[A] life without a struggle…is hardly a life worth living.”

The true gain is in the struggle for excellence, and “a life without such a goal is not really living but it is merely existing” [4].
